# Phylogeography of a semi-aquatic bug, *Microvelia horvathi* (Hemiptera: Veliidae): an evaluation of historical, geographical and ecological factors

**DOI:** 10.1038/srep21932

**Published:** 2016-02-29

**Authors:** Zhen Ye, Gengping Zhu, Jakob Damgaard, Xin Chen, Pingping Chen, Wenjun Bu

**Affiliations:** 1 College of Environmental Science and Engineering, Nankai University, 94 Weijin Road, Tianjin, 300071, China; 2 Institute of Entomology, College of Life Sciences, Nankai University, 94 Weijin Road, Tianjin, 300071, China; 3 Tianjin Key Laboratory of Animal and Plant Resistance, College of Life Sciences, Tianjin Normal University, 393 Binshui West Road, Tianjin 300387, China; 4 Natural History Museum of Denmark, Zoological Museum, Universitetsparken 15, 2100 Ø, Denmark; 5 Netherlands Biodiversity Centre Naturalis, 2300 RA Leiden, The Netherlands

## Abstract

Subtropical China is a centre of speciation and well known for its high biological diversity and endemism. To understand the impact of historical, geographical and ecological factors on the intraspecific lineage divergence of invertebrates, we examined these processes in a semiaquatic bug, *Microvelia horvathi* (Hemiptera: Veliidae). Three hypotheses were developed using ecological niche models (ENM). We tested these hypotheses using mitochondrial (COI + COII) and nuclear data (ITS1 + 5.8S + ITS2). The phylogenic analysis revealed a shallow divergence in mitochondrial data. Clade I was mostly confined to the northern region and clade II was nearly restricted to the southern region. The historical process of Pleistocene climatic fluctuations during the LGM promoted divergence, along with such geographical barriers as the Wuyi, Nanling and Xuefeng mountains and ecological factors of temperature and vegetation type, contributed to these shallow genetic divergences and helped maintain them. The north-south population differentiation probably occurred during the transition from LIG to LGM, with post-LGM population expansion. The results of genetic data were mostly consistent with the spatial predictions from ENM. Our study emphasizes the multiple effects influencing genetic population differentiation, and also contributes to our knowledge of the phylogeography of other aquatic organisms in subtropical China.

Understanding the historical and environmental factors that influence the genetic structure of populations is important for studying the mechanisms of adaptive divergence and speciation. In the Northern Hemisphere, patterns of contemporary population genetic structure of extant species have been greatly affected by Pleistocene climatic fluctuations, such as range expansion or isolation in glacial refugia^1^ and geographical features, such as spatial distance and geographical barriers^2^. Besides these two factors, Very recent environmentally mediated episodes (ecological factors) might also contribute to genetic differentiation^3^.

The Asian subtropics are a global biodiversity hotspot considered to be one of the most important areas of endemism for many organisms^4^. This region experienced repeated climate changes during the Pleistocene and also exhibited dramatic geographic heterogeneity^5^. In China, the subtropical zone ranges from the eastern Tibetan Plateau to the Pacific Ocean and from the Qingling Mountains-Huai River line (34° N) to the tropical south (20° N). Since Pleistocene climatic fluctuations in subtropical China were less extensive than in Western Europe and North America, and most areas were not covered by large ice sheets during the late Pleistocene^4^, it had been argued that homogenizing gene flow during the Pleistocene inhibited divergence among the populations^6^. However, in a few cases Pleistocene lineage diversification had been documented in some organisms^7^,^8^. This raised the question why some population experienced continuous gene flow while others experienced diversification. The study of demographic expansion over time also showed contradictory patterns in subtropical China. Some species, such as the fig-wasp, *Ceratosolen* sp.^9^ and the tree, *Castanopsis eyrei*^10^, revealed dramatic post-LGM expansion scenarios. Other species showed the opposite, for instance, the green-backed tit, *Parus monticolus*^11^ and another semi-aquatic bug, *Microvelia dougalsi douglasi*^12^ underwent unusual pre-LGM expansion. These apparently conflicting results suggested that subtropical China underwent complex Pleistocene climate changes throughout the last ice age cycles even though not covered by the ice sheet^13^, causing different responses by different organisms. In addition, geological tectonic uplift in subtropical China occurred in the late Miocene and Pliocene, resulting in relatively high mountain systems such as the eastern Himalayas and the mountains of South and Southwest China (the Hengduan, Wuyi, Nanling Mts.)^14^,^15^. These geographically heterogeneous mountain systems in subtropical China might have caused genetic diversification among populations adapting to the new stable environments, and mountainous areas also might have harboured many refugial populations in periods of adverse climatic conditions^13^, all of which led to the formation of new lineages/taxa and contributed to high genetic diversity.

Phylogeography has benefitted from recent developments in GIS technology, which now allows a more powerful investigation of the geographic components of genetic variation^16^. Ecological niche models (ENM) projects a model of past climatic conditions to explore the historic distribution of a species and the degree to which its distribution has changed^17^. This approach to generating a historical distribution is spatially explicit and independent from phylogeographical inference^18^. The use of ENM to explore past species distributions could supplement the limited invertebrate fossil record in subtropical China and, in combination with molecular data, enhance our understanding of underlying historical events and population dynamics (dispersal barriers, population size changes and localization of putative Pleistocene refugia) through time^16^.

Many previous phylogeographical studies have focused on plant and vertebrate species in subtropical China^10^,^19^. To reveal a more complete phylogeographical fauna pattern, common and widespread species need to be investigated, especially invertebrates. To understand the impact of Pleistocene climatic fluctuations, geographical barriers and ecological factors on the phylogeography of invertebrates, we examined these processes on a widespread subtropical semiaquatic bug, *Microvelia horvathi* Lundblad 1933, which belongs to the familiy Veliidae (Insecta: Hemiptera), and is found in the subtropics of China, Japan and Korea. This species usually lives on the quiet water surfaces of nearshore areas, including rain pools, ditches, swampy ground, or paddy fields^20^. Its distribution range is mostly endemic to subtropical China and restricted by geographical barriers (the Taihang, Qinling and Hengduan Mts.) (Fig. 1), suggesting that this small semi-aquatic insect might have a relatively low dispersal ability and be sensitive to climate fluctuations. Its distribution range includes a series of east-west mountain ranges (the Xuefeng, Nanling and Wuyi Mts.) (Fig. 1). Thus, gene flow among populations, especially populations on both sides of these mountains, is likely to be obstructed or slowered, and genetic differentiation is expected.

In this study, we first generated potential distribution maps of *M. horvathi* in the present, the LGM and the LIG. ENM projected that *M. horvathi* potential spaces contracted and were ultimately isolated in two mainly separated refugia (northern and southern refugia) where they underwent major divergence (Fig. 2, middle panel) during the LGM period. After that, the populations expanded from the separated refugia and came into contact with gene exchange, resulting in a largely continuous range in subtropical China exhibited in our current predicted space (Fig. 2, left panel). Based on the result of ENM, we derived three hypotheses: If the populations within these two refugia still had strong gene exchange with each other through secondary contact, counteracting the accumulated major divergence during the LGM, molecular data should reveal a relatively homogenizing genetic pattern between the northern and southern populations in this scenario (Hypothesis 1). Otherwise, if geographical barriers or ecological factors inhibited or slowed down the homogenizing gene flow between northern and southern populations, in this scenario a shallow genetic divergence pattern should be revealed between northern and southern populations in the molecular data (Hypothesis 2). Finally, since the ENM showed unsuitable LGM climate conditions and suitable current climate conditions (Fig. 2), we expected analyses of demographic history (mtDNA) to show that population expansion followed the same pattern (Hypothesis 3).

## Results

### Paleoclimate niche modeling reconstruction

A high AUC value was obtained from the current potential distribution (AUC = 0.987), indicating good predictive model performance. The current niche-predicted distribution of this species was generally similar to its actual distribution, with a potentially continuous range in subtropical China and a fragmentary range in southern Japan and Korea (Fig. 2). When projecting the current niche into historical climate conditions, the suitable climate space continued to stay *in situ* during the LIG period, but expanded moderately toward southwestern China and most parts of Japan and Korea (Fig. 2). When the ice age arrived (LGM period), under the CCSM model, the species’ potential range contracted greatly and was mainly located in two segregated refugia (the Sichuan Basin space and a space from northern Vietnam to northern Guangxi) (Fig. 2).

### Genetic polymorphism and phylogenetic analysis

**Figure 1:**
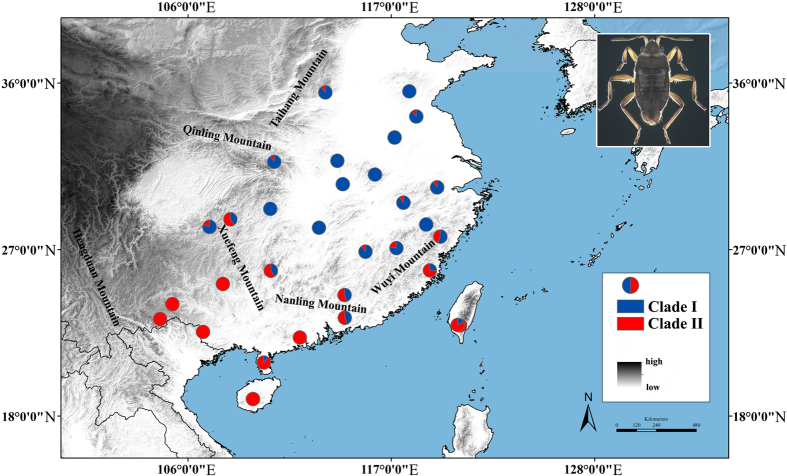
Sample size and halotype frequencies of two major clades of *M. horvathi,* designated clade I and II according to the molecular phylogeny in Fig. 3a. Frequencies of clade I and II haplotypes in each population are showed by the pie graph. Names of mountain ranges in the map were labelled. Figure was generated in ArcGIS 10 (Environmental Systems Research Institute).

For the mitochondrial data, 1405 bp of protein-coding regions of mitochondrial genome were obtained from 250 individuals, including sections of the COI (726 bp) and COII (679 bp) genes. Ninety unique haplotypes were derived among all individuals. The 96 polymorphic sites included 67 singleton variable and 29 parsimony informative sites. The nucleotide diversities and haplotype diversities ranged from 0.00057 to 0.00360 with an average of 0.00262, and 0.378 to 1.000 with an average of 0.845, respectively (Table 1). NJ analyses produced tree topology based on combined mitochondrial sequences (COI + COII) (Fig. 3a). The whole haplotypes were shallowly separated into two supported major clades, clade I and clade II. The haplotype frequencies of clade I and clade II were shown in a pie graph revealing that the geographical distributions of the two monophyletic clades were uneven (Fig. 1). Most haplotypes of clade I were located widely in the northern region of subtropical China, whereas most haplotypes of clade II were commonly found in the narrower southern region (Fig. 1). Interestingly, the intermediate populations (ZJTS, JXLN and GZYH) living between the northern and southern regions simultaneously possessed the haplotype diversities of both lineages. The network analysis based on all ninety haplotypes showed the same pattern, with two well-defined independent haplogroups (Figs 3b and 4a). In the haplotype network (Fig. 4a), h1, h12, h15 and h25 were the four most frequent haplotypes, characterizing 37.6%, 6%, 3.2% and 9.6% of individuals respectively. The h1 ancestral haplotype was mostly found in the northern region, whereas h12, h15 and h25 were the ancestral haplotypes found in the southern region (Table 1).

For the nuclear sequencing data, 1039 bp fragments were successfully obtained from 237 individuals, including ITS1, 5.8S and ITS2 genes. 19 unique haplotypes were derived among all individuals. The 19 polymorphic sites included 16 singleton variable and 3 parsimony informative sites. The nucleotide diversities and haplotype diversities ranged from 0 to 0.00123 with an average of 0.00055, and 0.000 to 0.833 with an average of 0.478, respectively (Table S1). The mitochondrial DNA structure of lineage differentiation in *M. horvathi* was not apparent in the nuclear data (Fig. 4b).

### Phylogeographic structure and hierarchical partitioning of genetic structure

SAMOVA showed a maximum value in *F*_CT_ of *K* = 2 (Fig. 5). The two groups corresponded genetically and geographically (i.e. north and south populations) in subtropical China. Zones of genetic discontinuities identified by Barrier 2.2 showed the potential geographical barriers associated with the genetic abruption (Fig. S1). The bold lines mainly divided the entire sampling region into a northern and a southern subregion, reflecting significant genetic/geographical isolation between them. Isolations between the northern and southern subregions (Fig. S1) were mostly consistent with the results of SAMOVA. The nuclear sequencing data showed much lower genetic diversities than the mitochondrial data, and revealed no distinct phylogeographical structure pattern (Fig. 4b). Therefore SAMOVA and Barrier analysis were not implemented in nuclear data.

AMOVA analysis showed a relatively significant genetic variance between the northern and southern groups in mitochondrial DNA data (48.45%, *P* < 0.05) (Table S2). However, the same analyses of nuclear DNA data attributed most genetic variance (83.69%) to differentiation among individuals within populations (Table S2). The Mantel tests revealed a significant positive relationship in the mitochondrial DNA (r = 0.5590, *P* < 0.001), but not in nuclear DNA (r = −0.0478, *P* = 0.3120) (Fig. S2a,b).

### Historical demographic changes in mitochondrial data

The negative values of Tajima’s *D*, Fu and Li’s D*, and the significance of Fu’s *F*s (Table 2) suggested a scenario of recent demographic expansion in the northern and southern populations. For the whole dataset, the negative values of D, D*, together with the significance of *F*s (Table 2) also suggested an overall past population expansion. Divergence time between clade I and clade II from the results of BEAST was moderately in keeping with the result of IMa2, which was estimated to be 0.051 Ma (95% HPD: 0.073–0.031 Ma) and 0.022 Ma (95% HPD: 0.033–0.016 Ma) separately (i.e. population divergence occurred during the LIG to LGM transition). From *t* = *τ*/2*u*, we estimated the expansion time ranged from approximately 12.75–25.50 Ka, and the Bayesian skyline demographic reconstructions showed a pattern of population growth through time in which the plots started from 15 Ka (i.e., population expansion occurred in the post-LGM period) (Fig. 3c).

### Ecological vicariance analyses

SEEVA analysis compared eight environmental variables between the sister lineages for *M. horvathi*. Three of eight comparisons were significant (Table S3). The phylogenetic splits between the north and south lineages showed strong divergence in terms of annual mean temperature (BIO1, D = 0.73*), minimum temperature of the coldest month (BIO6, D = 1.0*) and vegetation (D = 0.82*) (Fig. 6). Although *M. horvathi* depended on the water habitat for survival, the split between the north and south lineages only showed a slight divergence in annual precipitation (BIO12, D = 0.40) and precipitation in the driest month (BIO14, D = 0.41). The PCA-env results suggested the two different lineages might not occupy identical habitat, but the differences could simply be due to the differential availability of habitat in the different regions that they occupied (Fig. S3).

## Discussion

In this paper, we applied ENM to develop three hypotheses about genetic structure and population demography and tested the hypotheses by analyzing two genetic data sets in a phylogeographical framework. Below, we discuss these hypotheses in the light of our a priori expectations and the observed results.

A shallow divergence pattern between northern and southern populations was revealed by mitochondrial DNA, but no such pattern was found in nuclear DNA, which instead showed a widely shared ancestral haplotype (Fig. 4). Such discordances between mitochondrial and nuclear data might result from a variety of factors, such as incomplete lineage sorting of ancestral polymorphism, potentially sex-biased dispersal or the different evolutionary rates of different markers^10^. Firstly, if incomplete lineage sorting leads to the observed mitochondrial DNA pattern, we would anticipate a less shallow divergence pattern in the nuclear DNA, since the relatively larger effective population size would result in a longer sorting time^21^. However, analysis of the nuclear DNA data did not reveal any genetic divergence in the populations, suggesting that incomplete lineage sorting was not likely to be the main reason. Secondly, since sex-biased dispersive behavior influencing the phylogeographical structure had been studied in many animals^22^, we hypothesized that if females possessed native philopatry and males displayed high dispersal capacity, it would show that a significant IBD pattern detected in the mitochondrial DNA and a nonsignificant IBD pattern in nuclear DNA^23^. Interestingly, this hypothesis was in accordance with our IBD results (Fig. S2), which did not reject the possibility that a higher male dispersal ability was responsible for incongruence between mitochondrial DNA and nuclear DNA. However, there were no direct evidences in the literature for *M. horvathi* to testify whether the different sexes showed different dispersal capacity, which should be studied further through ecological and biological observations or experiments in the future. Thirdly, the reconstructed palaeodistribution from ENM prediction might show that the discordance patterns observed in mitochondrial DNA and nuclear DNA were most likely explained by the different evolutionary rates of these two markers. The ENM indicated a potentially largely continuous range in subtropical China during the LIG period with relatively high temperature than now^24^, but in the LGM period, the most suitable habitats contracted into two main refugia (the Sichuan Basin space and a space from northern Vietnam to northern Guangxi). Under the CCSM model, we suggested that the glacially-induced population isolation was sustained only long enough for divergence in mitochondrial DNA, which possesses a relatively fast mutation rate, and in a small effective population size, but not enough for divergence in the nuclear gene (ITS), with a slower evolutionary rate and a larger effective population size. Based on the molecular evidence, the divergence in mtDNA accumulated during the LGM had not entirely disappeared though the northern and southern populations came into contact with gene exchange during the LGM to current transition. Geographical barriers and ecological factors likely inhibited or slowed down the homogenizing gene flow between the northern and southern populations, which shaped the currently shallow divergence pattern. This accorded with Hypothesis 2 above.

**Figure 2:**
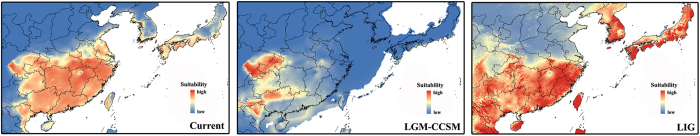
Hindcasting the current niche model (left panel) onto the LIG (right panel) and LGM (middle panel) periods in East Asia using Maxent. Niche model results were modified in ArcGIS 10 (Environmental Systems Research Institute).

It has been also demonstrated that mountains, as geographical barriers, played a key role in speciation and lineage differentiation by obstructing or slower migration/gene flow between populations^25^. Many previous studies had paid close attention to the effects of very large mountain systems such as the Hengduan and Qinling Mts., but less attention had been paid to the effect of the smaller mountain systems in subtropical China. In the Miocene and Pliocene, intensive tectonic activity had already begun in subtropical China^15^. Today, the north-south fauna dividing line in subtropical China was associated with a dramatic series of east-west trending mountain ridges reaching elevations of over 2000 m above sea level and differences between valleys and ridges extending 500 m above sea level in elevation. The Wuyi, Nanling and Xuefeng Mts were characteristic in this respect. The Wuyi and Nanling Mts. acted as protective barriers against the inflow of cold air from the northwest and retained warm moist air originating from the sea, resulting in a humid climate with high rainfall^26^. The Xuefeng Mountains ran northeast to southwest and acted as a protective barrier against different climate environments on the eastern edge of the Guizhou Plateau. These three east-west trending mountains were an important faunal dividing line between the Palaearctic Realm and the Oriental Realm^27^. We suggested that gene flow between the northern and southern populations was likely to be influenced by these mountains. Our molecular data confirmed hypothesis 2, showing that *M. horvathi* was genetically and geographically divided into north-south clades (Fig. 1, clade I and clade II). The haplotypes of clade I were widespread in the north and the haplotypes of clade II were mainly restricted to the narrow south areas. For example, some haplotypes were confined to the north or south, a haplotype h1 pattern which was found in 21 populations mostly distributed in the north of the mountains. A similar north-south geographical differentiation had been observed in previous studies, including the Chinese canopy tree *Eurycorymbus cavaleriei*^28^ and the small freshwater fish *Squalidus argentatus*^29^. Therefore, we speculated that the Wuyi, Nanling and Xuefeng Mts. might have acted as geographical barriers preventing or slowing down gene flow between the northern and southern populations, resulting in lineage differentiation especially during the inter-glacial periods. Further, these mountains areas commonly identified as biodiversity hotspots^30^ and acted as important refugia during Pleistocene glacial periods because of the extremely topographical complexity which could provide different suitable stable microclimates for many species to survive during the glacial period, harboring high diversities of haplotypes and lineages. Our molecular data supported this idea; the GZYH, GXME, JXLN, GDBL, ZJTS and FJSW populations of *M. horvathi* were entirely located in the Wuyi, Nanling and Xuefeng Mts., and occupied very high haplotype diversities relative to the other populations (e.g. *Hd*_GZYH_ = 0.844, *Hd*_GXME_ = 0.900, *Hd*_JXLN_ = 0.972, *Hd*_FJSW_ = 0.917), and all these populations also showed haplotype diversities both from clade I and clade II (Fig. 1). Thus, we suggested that these mountain areas were refugia for *M. horvathi* during the glacial period. In the LGM prediction under the CCSM model, one refugium was identified in northern Guangxi extending to northern Guangdong (areas of Nanling and Xuefeng Mts.) (Fig. 2). In conclusion, the Wuyi, Nanling and Xuefeng mountains not only as geographical barriers, obstructing or slowering gene flow between northern and southern populations and promoting to lineage differentiation, but also as potentially suitable areas during the glacial period, preserving the very high genetic diversities and diverse genetic lineages.

The geographical distribution of species was also affected by both historical and present-day ecological factors. Current ecological factors were clearly important in maintaining divergence of the genetic lineages of *M. horvathi*. This conclusion was based on the relatively strong coincidence between the northern and southern structural groups and zoogeographical fauna in subtropical China^27^, which revealed that the two groups occupied different climatic regimes (the Palaearctic Realm and Oriental Realm). In this case, the effects of ecological factors might be much more prominent for lineage differentiation. The Palaearctic Realm and Oriental Realm in subtropical China showed rather prominent difference in many environmental variables, such as temperature, precipitation, elevation and vegetation types. In the Palaearctic Realm of subtropical China, *M. horvathi* inhabited temperate deciduous forest and evergreen broad-leaved forest. In contrast, in the Oriental Realm, it mostly inhabited monsoon rain forest, evergreen broad-leaved forest and island monsoon rain forest, which were warmer and had more precipitation during the year. Among many present-day ecological factors, SEEVA analysis (Table S3, Fig. 6) showed that temperature and vegetation type greatly contributed to the phylogenetic split between the north and south lineages. The cooler northern region and warmer southern region might have easily affected the physiological activity of *M. horvathi*, gradually shaping the different temperature tolerance in the different lineages. However, the precipitation had less influence on the north-south lineage differentiation in subtropical China than expected, given that *M. horvathi* was a semi-aquatic species dependent on a water habitat for survival. It was reasonable to assume that *M. horvathi,* as a very small insect, only needed minor water microhabitats, and that large-scale precipitation might probably destroy microhabitats including small rain pools, ditches, and swampy ground. Therefore, the main ecological constraint on local lineages could be temperature, with the genetic differentiation resulting from Pleistocene climatic fluctuations being more or less maintained. However, to clarify how different lineages of *M. horvathi* adapted to local ecological niches, we still need to further explore the evidence, including studies of the function- related protein-coding genes.

**Figure 3:**
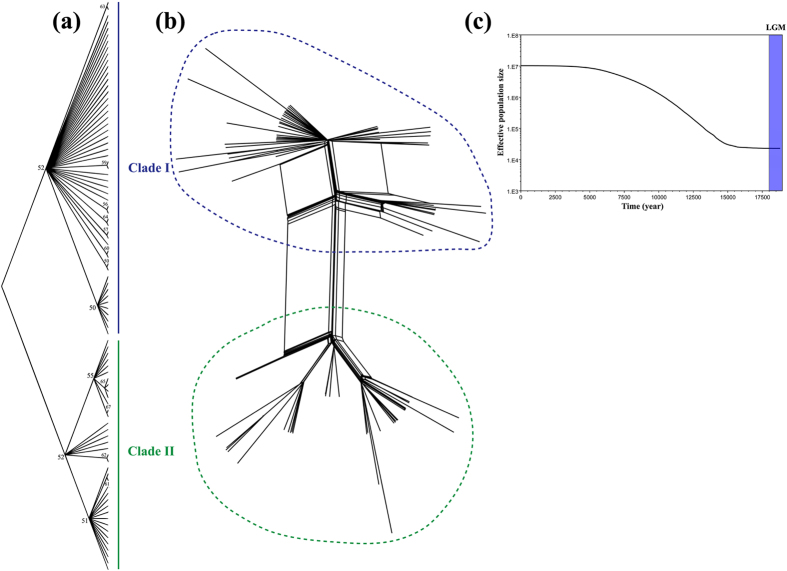
(**a**) Phylogenetic NJ tree analyses of all ninety haloptypes based on mitochondrial markers (COI + COII). Branches of above 50% bootstrap support are showed in number. (**b**) The Neighbor-Net algorithm. Clade I and clade II are shown in blue (at the top) and green (at the bottom), respectively. (**c**) Historical demographic trends of the whole data represented by Bayesian skyline plot (BSP) based on mitochondrial data. X axis is the time scale before present. Y axis is the estimated effective population size. Estimates of means are joined by a solid line. LGM represents Last Glacial Maximum.

**Figure 4:**
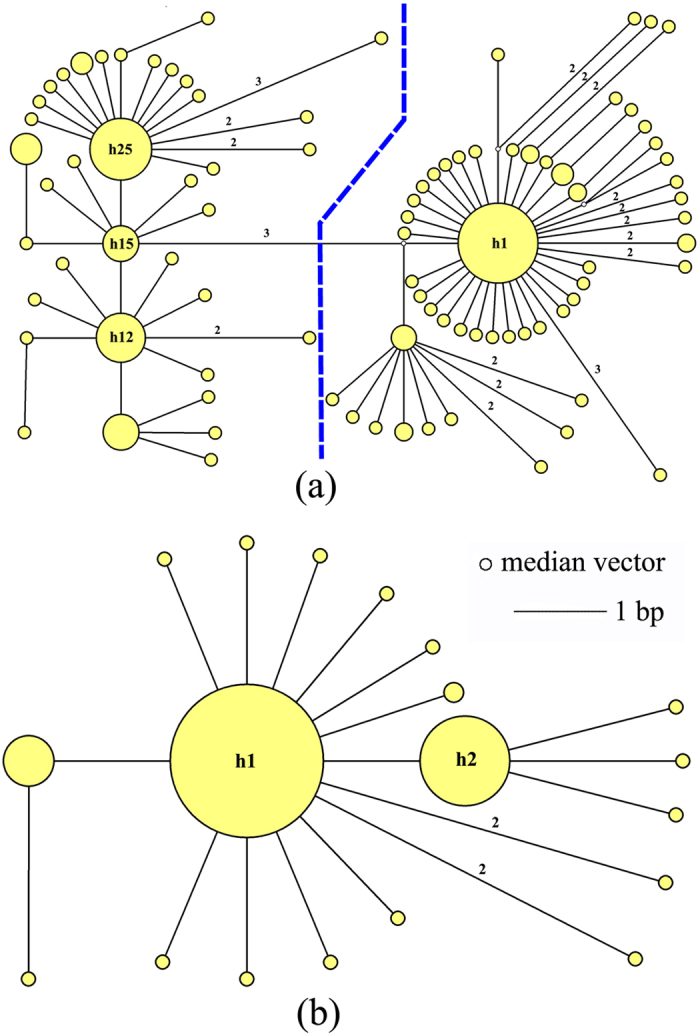
Median joining haplotypes network constructed using Network. Haplotype circle size denotes the number of sampled individuals. Numbers of base pair changes (no number = 1 bp) are given. (**a**) Based on mitochondrial data. (**b**) Based on nuclear data.

**Figure 5:**
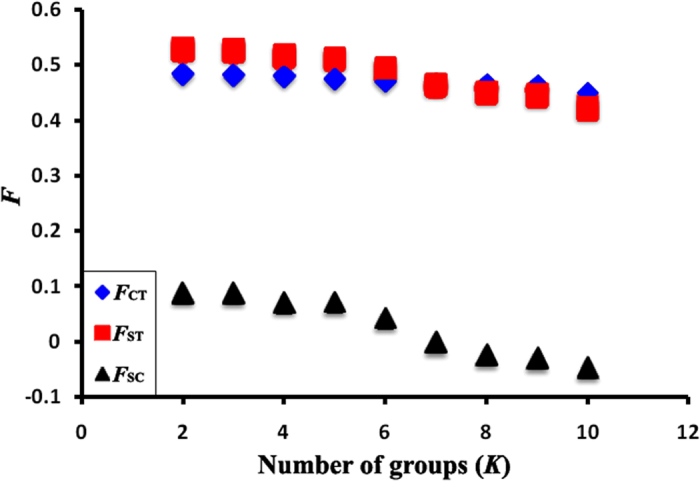
Values of fixation indices (*F*) as a function of number of groups (*K*) based on mitochondrial data. *F*_CT_ is the differentiation between groups, *F*_ST_ is the differentiation between populations among groups, and *F*_SC_ is the differentiation between populations within groups.

**Figure 6:**
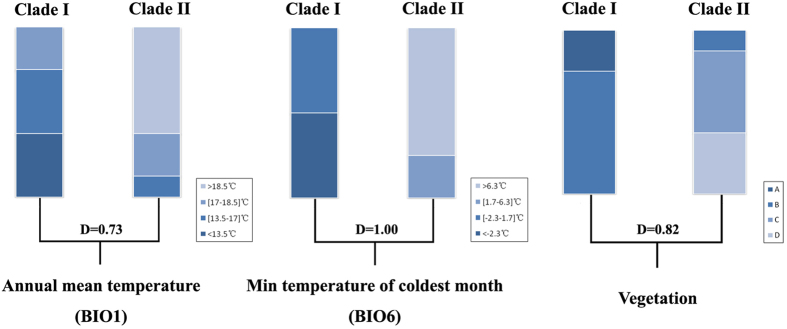
The results of the spatial evolutionary and ecological vicariance analysis (SEEVA) of *M. horvathi* using annual mean temperature (BIO1), min temperature of coldest month (BIO6) and vegetation type as four qualitative or quantitative section states. The blue scale of the histograms represents the four different states. Vegetation: A, temperate deciduous forest; B, evergreen broad leaved forest; C, monsoon rain forest/evergreen broad leaved forest; D, island monsoon rain forest.

Estimating divergence time derived from the mitochondrial data in BEAST and IMa2 indicated that diversification of *M. horvathi* was fairly recent. The north-south differentiation probably occurred during the transition from the LIG to the LGM. In the MJ haplotypes network, four starlike shapes in the south region and one relatively large starlike shape in the north region (Fig. 4a) suggested that *M. horvathi* experienced more population expansion events in the south region than in the north region. The molecular result of neutrality tests (Table 2) and the Bayesian skyline plot (BSP) (Fig. 3c) all indicated that historical population expansion happened after the LGM period. These results were in line with the spatial prediction of ENM (Hypothesis 3) (Fig. 2). From the LIG to LGM period, when the weather gradually became increasingly dryer and colder, potential spaces for *M. horvathi* contracted and were gradually isolated into two largely separate northern and southern refugia (Fig. 2), after which populations in the isolated refugia had less gene exchange and accumulated major divergence. After the LGM period, when the weather turned warm and moist, the two formerly separate groups expanded and a significantly suitable range increased into the current predicted space (Fig. 2). These two independent hindcasting methods (phylogeograpic analysis and ENM) achieved a similar historical demographic change pattern. Interestingly, this result was contradictory to the previous phylogeographic studies in *Microvelia douglasi douglasi*^12^, although these two species both belong to the same genus. We speculated that the main reason for the contradictory patterns might be differences in the ecologic requirements of these two species. *M. douglasi douglasi* was widely distributed in the east and southeastern Asia today, but *M. horvathi* was mainly confined to subtropical China. The wide distributional range meant that *M. douglasi douglasi* exhibited much higher genetic diversity, with the potential to adapt to diverse environments easily. Secondly, in the potential LGM distribution predicted by ENM, *M. dougalsi douglasi* inhabited connected and relatively large areas in subtropical China and tropical Asia including Vietnam and Indonesia, while *M. horvathi* only inhabited segregated, narrow and discontinuous areas in subtropical China (Fig. 2). Therefore, we proposed that *M. horvathi* might be less adapted to the whole range of local environments in subtropical China than *M. douglasi dougalsi*, with relatively lower ecological tolerance, and was particularly sensitive to population changes driven by Pleistocene climatic fluctuations.

## Methods

### Sample collection and DNA sequencing

We selected 31 sampling sites for *M. horvathi* across the natural distributional area, which largely covered the whole distributional range of this species in China (Fig. 1, Fig. S1). Distribution maps were generated by the software ArcGIS 10 (ESRI, Redlands, CA, USA). In each population, three to ten (mean of eight) mature males or females were collected. All the materials were deposited in the College of Life Sciences at Nankai University (Tianjin, China). Genomic DNA was extracted from the entire body excluding the genitalia. A total of 250 individuals were sequenced for mitochondrial markers (COI + COII) and nuclear markers (ITS1 + 5.8S + ITS2). Polymerase chain reactions (PCR) were performed using specific primers following Ye *et al.* (2014). The PCR procedure for COI, COII and nuclear markers (ITS1 + 5.8S + ITS2) included an initial denaturation at 94 °C for 2 minutes, followed by 31–33 cycles of 30 seconds at 92 °C, 30 seconds at 48–52 °C and 1 minute at 72 °C, ending with a final extension at 72 °C for 8 minutes. The chromatograms of sequences were unimodal, which excluded pseudogene disturbance. Sequences were visually proofread and aligned in Bioedit 7.1 software^31^.

### Paleoclimate niche modeling reconstruction

A total of 47 occurrence records were obtained for niche modeling, including 33 records from the alcohol preserved specimens and 14 records from pinned specimens in Nankai University or the published literature. We chose the seven bioclimatic variables from the WorldClim database (http://www.worldclim.org/) that were thought to most likely restrict semi-aquatic bugs distribution^12^, namely, annual mean temperature (BIO1), mean diurnal temperature range (BIO2), maximum temperature of the warmest month (BIO5), minimum temperature of the coldest month (BIO6), annual mean precipitation (BIO12), precipitation of the wettest month (BIO13), and precipitation of the driest month (BIO14). All the variables were derived from the WorldClim data center at a resolution of 2.5-arc. We used maximum entropy implemented in Maxent (version 3.3.3 k^32^) to estimate niches in environmental dimensions. Analysis was run using default program conditions. Area Under Curve (AUC) of the Receiver Operating Characteristic (ROC) plot was used for model evaluation. For hindcasting the effect of Pleistocene climatic fluctuations, the current native niche models were calibrated using the above environmental variables and then transferred onto the reconstructed climatic conditions during the LGM and LIG periods. The Community Climate System Model 3 (CCSM 3) for LGM conditions was used.

### Genetic polymorphism and phylogenetic analysis

We estimated genetic diversities by the number of polymorphic sites (*S*), haplotype distribution (Hap), number of haplotypes (Nhap), haplotype diversity (*Hd*), and nucleotide diversity (*π*), which were all calculated in DNASP 4.0^33^. We used the uncorrected *p* distances to reconstruct an unrooted NJ tree to elucidate the phylogenetic relationships among mitochondrial haplotypes of *M. horvathi* using PAUP* 4.0b10^34^. Statistical support for phylogenetic grouping was assessed by 1000 bootstrap replicates. To further investigate the relationships among unique haplotypes, we constructed unrooted networks using two methods: the neighbor-net algorithm with SplitsTree 4.6^35^, and a median-joining method^36^ constructed with default settings in Network 4.6.1.3 (Fluxus Technology, Suffolk, UK).

### Phylogeographic structure and hierarchical partitioning of genetic structure

The SAMOVA 1.0^37^ program was used to simulate 100 simulated annealing processes for *K* = 2 to 10. The potential geographical zones associated with genetic discontinuities across the entire sample region were further investigated using Monmonier’s algorithm implemented in Barrier 2.2^38^. Mitochondrial DNA identified two major clades, which were apparently consistent with a north-south geographic partition (Figs 1 and 3a). We assessed the hierarchical partitioning of genetic structure between the two groups (north and south) resulting from SAMOVA analysis in each marker using analysis of molecular variance (AMOVA) in Arlequin 3.5^39^. The statistical significance of variance components was assessed using 10000 random permutations. Additionally, for testing an isolation-by-distance model, the Mantel test was performed in IBDWS^40^ for each genetic marker using 1000 randomizations.

### Historical demographic changes based on mitochondrial data

We used three neutrality tests, the Tajima’s *D*, Fu and Li’s D* and Fu’s Fs tests, implemented in DNASP 4.0^33^ or Arlequin 3.5^39^, to detect deviations from the mutation-drift equilibrium that would be indicative of changes in historical demography and natural selection. We used BEAST 1.8.2^41^ to estimate divergence time for the mitochondrial lineages obtained. The best fit nucleotide substitution model GTR + G + I was estimated by Modeltest 3.7^42^. Divergence time was estimated with an uncorrelated lognormal relaxed clock with the mutation rate 0.4–0.8%/Myr for *M. horvathi*^43^. We adopted one year as seven generation for *M. horvathi*^44^, using a coalescent tree prior model with the chains run for 100 million generations, checked to make sure the ESS was more than 200 and then discarded the first 10% as burn-in. We also estimated divergence time using the isolation-with-migration model implemented in IMa2^45^. The HKY model and 0.25 as inheritance scalar were used. We set the upper prior bounds to 80 for the population size (q), 32 for the divergence time (t) and 0.125 for the migration rate (m). Final runs consisted of 1 × 10^8^ steps with a burn-in of 1 × 10^7^ steps, with the lowest ESS among the parameters at least above 50.

Then two methods were used to estimate population expansion time. One approach used *t* = *τ*/2*u* (where *τ* is the crest of mismatch distribution, *u* is the mutation rate per generation for the whole sequence, and *t* is the expansion time in number of generations; the value *u* was calculated using the formula *u* = *μk* (*μ* is the mutation rate per nucleotide and *k* is the number of nucleotides). The other approach used a Bayesian coalescent-based method (Bayesian Skyline Plot, BSP) in BEAST 1.8.2. A relaxed uncorrelated lognormal molecular clock was applied. We ran the chains for 100 million generations, and checked to make sure the ESS was more than 200, after which the first 10% were discarded as “burn-in”.

### Ecological vicariance analyses

We used two approaches to test ecological divergences between two phylogenetic lineages of *M. horvathi*. First, we tested the correlations between the ecological divergences and the phylogenetic splits using spatial evolutionary and ecological vicariance analysis (SEEVA^46^). We extracted seven environmental variables (BIO1, BIO5, BIO6, BIO12, BIO13, BIO14 and Elevation) for each sampling site in ArcGIS (ESRI, Redlands, CA, USA), and primary GIS data layers for temperature and precipitation were obtained from the WorldClim data (http://www.worldclim.org/). Quantitative variables (temperature, elevation and precipitation) were scored as ordered continuous data, while the qualitative variables (vegetation type) were treated as non-ordered categorical data. We divided these variables into four states according to the default setting in SEEVA. We investigated the correlations between ecological shifts and the phylogenetic splits using Fisher’s exact tests and divergence indices (D). Second we used PCA-env function in the R script^47^ to measure niche overlaps in gridded environmental space between the two lineages^48^. Observed densities for each region were corrected in light of the availability of environmental space using kernel density functions. Niche overlap was measured along gradients of a multivariate analysis (i.e. principal component analysis, PCA), and the statistical tests of niche equivalency and similarity^45^ were computed from the density estimations of taxa in environmental space. Similarity was quantified using Schoener’s *D*, with values ranging from 0 to 1, or from more to less similar respectively.

## Additional Information

**How to cite this article**: Ye, Z. *et al.* Phylogeography of a semi-aquatic bug, *Microvelia horvathi* (Hemiptera: Veliidae): an evaluation of historical, geographical and ecological factors. *Sci. Rep.* **6**, 21932; doi: 10.1038/srep21932 (2016).

## Supplementary Material

Supplementary Information

## Figures and Tables

**Table 1:** Nucleotide polymorphism in each geographic population.

COI + COII	Lat.	Long.	Sample size	*S*	Hap	*Hd*	*π*
Northern populations							
AHBB	33°3′30″	117°10′41″	9	6	**h1**(5), h2, **h3**, h4, h5	0.722	0.00095
AHYX	31°3′34″	116°6′52″	10	6	**h1**(6), **h6**, **h7**, h8, h9	0.667	0.00085
FJSW	27°5′5″	117°16′25″	9	10	**h1**(3), **h6**, **h7**, h10, h11, **h12**, **h13**	0.917	0.00225
GDBL	23°19′10″	114°28′44″	9	16	**h1**(2), **h15**, h17, h18, h19, h20, h21, h22	0.972	0.00360
GXME	25°51′27″	110°28′38″	5	10	**h12**, **h29**(2), h32, h33,	0.900	0.00356
GZSY	28°13′54″	107°9′38″	10	10	**h1**(6), **h25**, **h29**, h42, h43	0.667	0.00195
GZYH	28°38′16″	108°17′22″	10	12	**h1**(2), **h36**(4), h44, h45, h46, h47	0.844	0.00313
HNBB	35°30′6″	113°25′41″	9	10	**h1**(6), **h12**, h49, h50	0.583	0.00170
HNXY	31°48′8″	114°4′36″	8	4	**h1**(6), **h51**, h52	0.464	0.00071
HBSN	31°44′46″	110°39′38″	10	9	**h1**(7), **h6**, **h36**, **h53**	0.533	0.00128
HBWH	30°32′25″	114°22′20″	9	6	**h1**(6), h54, h55, h56	0.583	0.00095
HNCS	28°10′57″	113°4′59″	5	3	**h1**(3), **h57**, h58	0.700	0.00085
HNZJ	29°12′22″	110°26′44″	10	4	**h1**(8), **h3**, h59	0.378	0.00057
JSXY	34°11′42″	118°20′52″	9	10	**h1**(5), **h12**, h60, h61, h62	0.722	0.00158
JXLN	24°32′40″	114°27′53″	9	10	**h1**, **h12**, **h15**, **h25**(2), h63, h64, h65, h66	0.972	0.00265
JXML	29°32′18″	117°39′10″	10	13	**h1**(3), **h7**, **h15**, h67, h68, h69, h70, h71	0.933	0.00196
JXSX	26°53′21″	115°35′54″	10	9	**h1**(6), **h13**, **h28**, h72, h73	0.667	0.00128
SDMY	35°33′23″	117°58′19″	7	7	**h1**(3), **h7**, h74, h75, h76	0.857	0.00169
ZJLA	30°21′53″	119°28′41″	10	11	**h1**(7), **h12**, h85, h86	0.533	0.00168
ZJSC	28°21′38″	118°53′9″	8	7	**h1**(5), **h51**, h87, h88	0.643	0.00125
ZJTS	27°42′11″	119°38′55″	9	9	**h1**(3), **h25**(3), **h57**, h89, h90	0.833	0.00261
Southern populations							
FJYT	25°52′42″	119°5′10″	4	8	**h12**, h14, **h15**, h16	1.000	0.00285
GDLZ	20°53′59″	110°5′48″	9	12	**h12**(2), h23, h24, h25, h26, h27, h28, h29	0.972	0.00233
GDNJ	22°14′56″	112°2′49″	6	6	**h15**(2), **h18**, h22, h23, h24, **h25**, h26, h27, **h28**	0.800	0.00157
GXNG	22°33′19″	106°48′21″	8	10	**h12**, **h25**, h34, h35, **h36**, h37, h38, h39	1.000	0.00241
GZML	25°8′49″	107°52′54″	9	7	**h12**(3), **h15**, **h28**, **h29**(2), h40, h41	0.889	0.00154
HNMY	18°55′35″	109°30′32″	2	2	h48, **h51**	1.000	0.00142
TWGX	22°54′41″	120°42′59″	8	6	**h12**, **h15**, **h25**(3), H77, h78, h79	0.893	0.00127
TWMN	22°55′51″	120°35′23″	6	7	**h1**, **h25**(4), h80	0.600	0.00180
YNBM	24°3′32″	105°8′43″	10	6	**h15**, **h25**(4), **h29**(2), h81, h82, h83	0.844	0.00127
YNXC	23°14′54″	104°28′43″	3	3	**h12**, **h25**, h84	1.000	0.00142

*S*, number of segregating sites; Hap, haplotypes’ distribution; *Hd*, haplotype diversity; *π*, nucleotide diversity. **Halotypes** had been found in two or more localities; the number in brackets indicates how many times a haplotype was observed at a particular locality.

**Table 2:** Nucleotide polymorphism and Neutrality tests in defined groups and whole dataset based on mitochondrial data.

Parameter	Northern populations	Southern populations	Whole set
Sample size	185	65	250
*S*	78	38	96
Nhap	60	30	90
*Hd*	0.745	0.899	0.845
*π*	0.00195	0.00182	0.00262
Tajima’s *D*	−2.46357**	−2.23081**	−2.33707*
Fu’s Fs	−26.47381***	−26.70583***	−25.65535***
Fu and Li’s D*	−7.88953**	−5.04140**	−8.81400**

*S*, number of segregating sites; NHap, number of haplotypes; *Hd*, haplotype diversity; *π*, nucleotide diversity.

*P < 0.05; **P < 0.02; ***P < 0.001.
