# Supplementary material for: Phylogeography of a semi-aquatic bug, Microvelia horvathi (Hemiptera: Veliidae): an evaluation of historical, geographical and ecological factors
Source: Sci Rep. 2016 Feb 29;6:21932. doi: 10.1038/srep21932 (PMC4770413; doi:10.1038/srep21932)

**Phylogeography of a semi-aquatic bug, *Microvelia horvathi* (Hemiptera: Veliidae): an evaluation of historical, geographical and ecological factors**

Zhen Ye^1, 2^, Gengping Zhu^3^, Jakob Damgaard^4^, Xin Chen^2^, Pingping Chen^5^, Wenjun Bu^2**^

1 *College of Environmental Science and Engineering,* *Nankai University, 94 Weijin Road, Tianjin, 300071, China*.

2 *Institute of Entomology, College of Life Sciences, Nankai University, 94 Weijin Road, Tianjin, 300071,China.*

3 *Tianjin Key Laboratory of Animal and Plant Resistance,* *College of Life Sciences, Tianjin Normal University, 393 Binshui West Road, Tianjin 300387, China.*

4 *Natural History Museum of Denmark, Zoological Museum, Universitetsparken 15, 2100 Ø, Denmark*.

5 *Netherlands Biodiversity Centre Naturalis, 2300 RA Leiden, The Netherlands.*

** Correspondence: Wenjun Bu, Fax: +86-22-23408957, Institute of Entomology, College of Life Sciences, Nankai University; 94 Weijin Road, Tianjin, 300071, China. E-mail: wenjunbu@nankai.edu.cn

**Table S1** Nucleotide polymorphism in each population with geographical coordinates. *S*, number of segregating sites; Hap, haplotypes’ distribution; *Hd*, haplotype diversity; *π*, nucleotide diversity (haplotypes in bold have been found in two or more localities; the number in brackets indicates how many time a haplotype has been observed at a particular locality).

| **ITS1+5.8S+ITS2** | **lat.** | **Long.** | **Sample size** | ***S*** | **Hap** | ***Hd*** | ***π*** |
| --- | --- | --- | --- | --- | --- | --- | --- |
| **North populations** |  |  |  |  |  |  |  |
| AHBB | 33°3'30" | 117°10'41" | 10 | 1 | **h1**(7), **h2**(3) | 0.467 | 0.00045 |
| AHYX | 31°3'34" | 116°6'52" | 9 | 3 | **h1**(4), **h2**(4)**,** h3 | 0.667 | 0.00096 |
| FJSW | 27°5'5" | 117°16'25" | 9 | 1 | **h1**(5), **h2**(4) | 0.556 | 0.00053 |
| GDBL | 23°19'10" | 114°28'44" | 10 | 3 | **h1**(7), **h6**, h7, h8 | 0.533 | 0.00073 |
| GXME | 25°51'27" | 110°28'38" | 8 | 1 | **h1**(5), **h2**(3) | 0.536 | 0.00052 |
| GZSY | 28°13'54" | 107°9'38" | 8 | 3 | **h1**(2), **h2**(4), **h4**, h9 | 0.750 | 0.00103 |
| GZYH | 28°38'16" | 108°17'22" | 9 | 1 | **h1**(7), **h2**(2) | 0.389 | 0.00037 |
| HNBB | 35°30'6" | 113°25'41" | 10 | 3 | **h1**(4), **h2**(5), h12 | 0.644 | 0.00092 |
| HNXY | 31°48'8" | 114°4'36" | 7 | 2 | **h1**(5), **h2**, h13 | 0.524 | 0.00055 |
| HBSN | 31°44'46" | 110°39'38" | 6 | 1 | **h1**(5), **h2** | 0.333 | 0.00032 |
| HBWH | 30°32'25" | 114°22'20" | 9 | 3 | **h1**(5), **h2**(2), h10, h11 | 0.694 | 0.00091 |
| HNCS | 28°10'57" | 113°4'59" | 5 | 1 | **h1**(3), **h2**(2) | 0.600 | 0.00058 |
| HNZJ | 29°12'22" | 110°26'44" | 9 | 2 | **h1**(6), **h2**(2), h14 | 0.556 | 0.00059 |
| JSXY | 34°11'42" | 118°20'52" | 7 | 2 | **h1**(4), **h2**(2), h15 | 0.667 | 0.00082 |
| JXLN | 24°32'40" | 114°27'53" | 8 | 1 | **h1**(7), **h6** | 0.250 | 0.00024 |
| JXML | 29°32'18" | 117°39'10" | 10 | 0 | **h1**(10) | 0.000 | 0.00000 |
| JXSX | 26°53'21" | 115°35'54" | 9 | 5 | **h1**(4), **h2**, **h6**, h16, h17, h18 | 0.833 | 0.00123 |
| SDMY | 35°33'23" | 117°58'19" | 5 | 1 | **h1**(4), **h2** | 0.400 | 0.00038 |
| ZJLA | 30°21'53" | 119°28'41" | 10 | 1 | **h1**(6), **h2**(4) | 0.533 | 0.00051 |
| ZJSC | 28°21'38" | 118°53'9" | 10 | 0 | **h1**(10) | 0.000 | 0.00000 |
| ZJTS | 27°42'11" | 119°38'55" | 6 | 1 | **h1**(5), h19 | 0.333 | 0.00032 |
| **South populations** |  |  |  |  |  |  |  |
| FJYT | 25°52'42" | 119°5'10" | 4 | 2 | **h1**(2), **h4**, h5 | 0.833 | 0.00096 |
| GDLZ | 20°53'59" | 110°5'48" | 9 | 1 | **h1**(8), **h6** | 0.222 | 0.00021 |
| GDNJ | 22°14'56" | 112°2'49" | 6 | 0 | **h1**(6) | 0.000 | 0.00000 |
| GXNG | 22°33'19" | 106°48'21" | 8 | 1 | **h1**(6), **h6**(2) | 0.429 | 0.00041 |
| GZML | 25°8'49" | 107°52'54" | 7 | 1 | **h1**(3), **h6**(4) | 0.571 | 0.00055 |
| HNMY | 18°55'35" | 109°30'32" | 1 | – | **h1** | – | – |
| TWGX | 22°54'41" | 120°42'59" | 9 | 0 | **h1**(9) | 0.000 | 0.00000 |
| TWMN | 22°55'51" | 120°35'23" | 6 | 0 | **h1**(6) | 0.000 | 0.00000 |
| YNBM | 24°3'32" | 105°8'43" | 10 | 1 | **h1**(8), **h6**(2) | 0.356 | 0.00034 |
| YNXC | 23°14'54" | 104°28'43" | 3 | 1 | **h1**(2), **h6** | 0.667 | 0.00064 |

**Table S2** Hierarchical analyses of molecular variance for *M. horvathi*

| **Genetic marker** | **Regional subdivisions** | **Source of variation** | **Variance explained** | ***P*** | **Fixation**  **index** |
| --- | --- | --- | --- | --- | --- |
| **mtDNA** | Northern & Southern groups | Among group | 48.45 | 0.00 | 0.484 |
|  |  | Among populations within groups | 4.54 | 0.00 | 0.088 |
|  |  | Within populations | 47.01 | 0.00 | 0.529 |
| **nrDNA** | Northern & Southern groups | Among group | 12.13 | 0.00 | 0.121 |
|  |  | Among populations within groups | 4.18 | 0.03 | 0.047 |
|  |  | Within populations | 83.69 | 0.00 | 0.163 |

mtDNA, mitochondrial DNA; nrDNA, nuclear DNA

**Table S3** Index of divergence (D) from spatial evolutionary and ecological vicariance analysis (SEEVA) for *Microvelia horvathi* using environmental variables. The clade I populations including AHBB, AHYX, HNCS, HNZJ, HBWH, HNXY, SDMY and ZJSC; The clade II populations including GDNJ, GXNG, GZML, HNMY, YNBM, YNXC and TWGX.

| **Clade I**  **VS**  **Clade II** | Annual mean temperature (BIO1) | Max temperature of warmest month (BIO5) | Min temperature of coldest month (BIO6) | Annual precipitation (BIO12) |
| --- | --- | --- | --- | --- |
|  | **0.73** | 0.43 | **1.00** | 0.40 |
|  | Precipitation of wettest month (BIO13) | Precipitation of driest month (BIO14) | Elevation | Vegetation |
|  | 0.25 | 0.41 | 0.33 | **0.82** |

Features with significant D-values > 0.50 are listed in bold face.

**Fig S1** Zones of genetic discontinuities. Barriers were retained under the majority-rule criteria according to their importance based on mitochondrial data. Figure was generated in ArcGIS 10 (Environmental Systems Research Institute) and Barrier 2.2^35^.


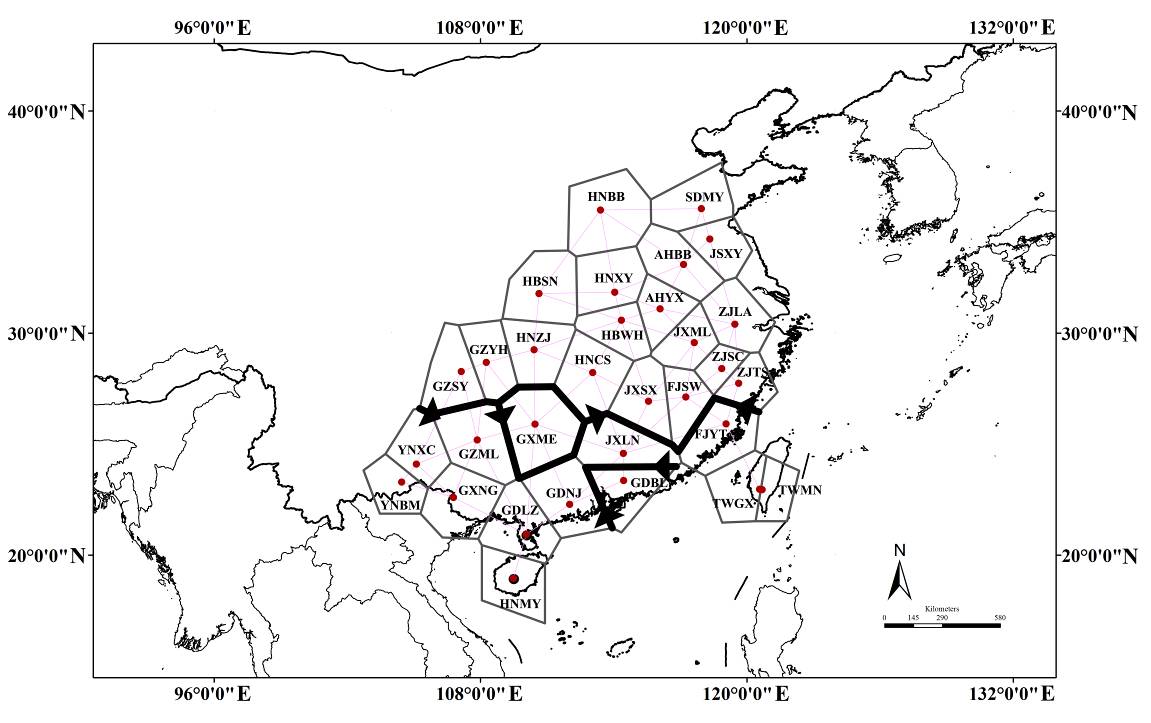


**Fig. S2** Scatter plot showing the relationship between genetic distances (*Фst*) and geographical distances (km). (a) Based on mitochondrial data. (b) Based on nuclear data.


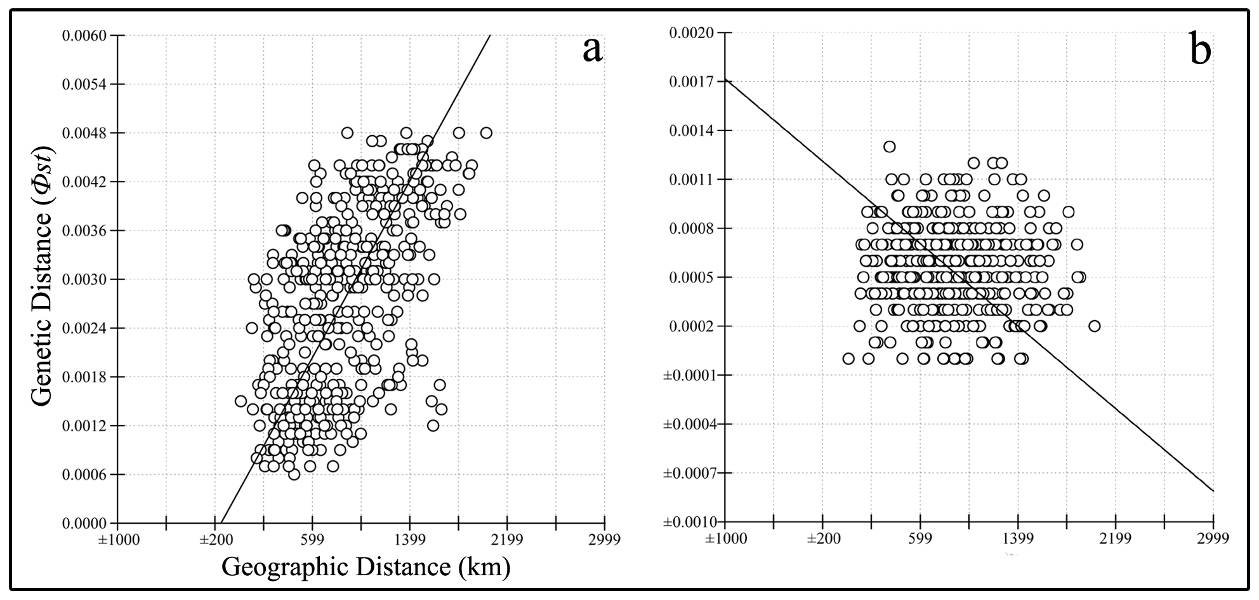


**Fig S3** Pairwise comparison of niches in climatic space (PCA-env) of two lineages in the *M. horvathi*. Upper left and upper right plots illustrate the niches of the two lineages compared; density of the occurrences of each lineage by cell is grey-shaded; solid and dashed contour lines illustrate 100% and 50% of the available environmental space, respectively. Lower left plot shows the contribution of the bioclimatic variables (i.e. BIO1, BIO2, BIO5, BIO6, BIO12, BIO13, BIO14) on the two axes of the principal components analysis and the explanatory power of the two main axes. Red diamonds indicate the position of the observed niche overlap.


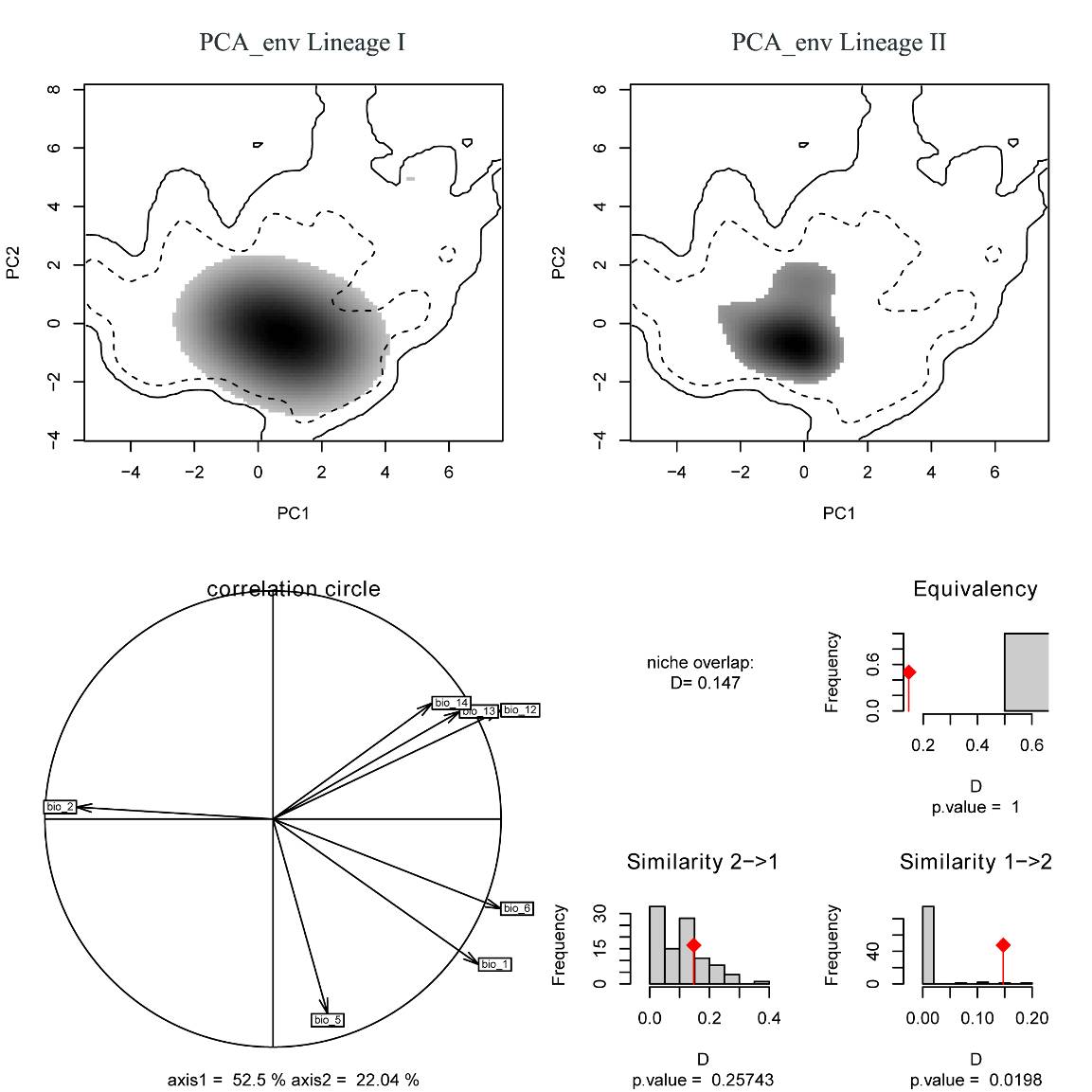

Supplement: Supplementary Information [file srep21932-s1.doc]
